# Mailuoshutong pill for varicocele-associated male infertility—Phytochemical characterisation and multitarget mechanism

**DOI:** 10.3389/fphar.2022.961011

**Published:** 2022-09-09

**Authors:** Dongfang Lv, Yun Ji, Qian Zhang, Zhuozhuo Shi, Tengfei Chen, Chao Zhang, Xiangyun Wang, Taotao Ren, Zhaowang Gao, Chongfu Zhong

**Affiliations:** ^1^ College of First Clinical Medicine, Shandong University of Traditional Chinese Medicine, Jinan, China.; ^2^ Affiliated Hospital of Shandong University of Traditional Chinese Medicine, Jinan, China.

**Keywords:** mailuoshutong pill, varicocele-associated male infertility, phytochemical characterisation, multitarget mechanism, network analysis, pharmacological experiments

## Abstract

**Background:** Varicocele (VC) is a relatively common and treatable cause of male infertility. Mailuoshutong pill (MLST), a traditional Chinese patent medicine, is widely used for treating varicose vein disease, but the underlying mechanism of MLST on varicocele-associated male infertility is unclear.

**Objective:** To reveal the phytochemical characterisation and multitarget mechanism of MLST on varicocele-associated male infertility.

**Methods:** The components in MLST were determined using UHPLC-MS/MS. Through network analysis, we constructed the “Drug-Components-Targets-Disease” network and predicted the potential biological functions and signaling pathways of MLST. Finally, the therapeutic effects and potential mechanisms of MLST were discovered by pharmacological experiments.

**Results:** By network analysis, the “Drug-Components-Targets-Disease” network was constructed, 62 components such as apigenin, limonin, kaempferol, and obacunoic acid may be the main active components of MLST for varicocele-associated male infertility, 28 targets such as VEGFA, PIK3CA, AKT1, and MTOR are considered as hub targets, signaling pathways such as HIF-1, Estrogen, PI3K/Akt, and mTOR may be key pathways for MLST against varicocele-associated male infertility. Through pharmacological experiments, we found that MLST ameliorated VC-induced testicular atrophy. Further histomorphology showed that MLST reduced VC-induced damage to testicular spermatogonia and seminiferous tubule, while MLST reduced ROS and MDA levels and increased antioxidant enzymes (GSH, GSH-Px, SOD, and CAT) levels. TUNEL staining and immunofluorescence showed that MLST reduced VC-induced apoptosis in testicular tissue, decreased BAX, and increased BCL2. Western blot results showed that MLST decreased the phosphorylation of PI3K, AKT, and mTOR proteins, and decreased the expression of HIF1α.

**Conclusion:** The phytochemical characterisation and multitarget mechanism of MLST on varicocele-associated male infertility were discovered using network analysis and pharmacological experiments. We verified that MLST can inhibit the activation of the PI3K/Akt/mTOR signaling pathway, reduce the expression of HIF1α, and further attenuate VC-induced oxidative stress and apoptosis in the testis. These findings provide evidence for the therapeutic role of MLST in varicocele-associated male infertility.

## Introduction

Varicocele (VC), one of the leading causes of male infertility, is characterized by abnormally enlarged veins within the pampiniform plexus (Jensen et al., 2017). Venous valve insufficiency and venous reflux disorder are currently recognized as the causes of VC (Silay et al., 2019). According to early reports, VC affects 15% of men, while 35%–40% of infertile men are affected (Agarwal et al., 2020; Gill et al., 2021). Studies have also shown that men with VC experience testicular atrophy and abnormal semen quality to varying degrees and that the negative effects of VC on spermatogenic function are progressive (Santana et al., 2020). Although the pathophysiological mechanisms of male infertility caused by VC have not been elucidated, current studies have focused on elevated local testicular temperature, oxidative stress, metabolite accumulation, and hormonal disorders (Razi et al., 2021). Varicocelectomy, the primary treatment for varicocele-associated male infertility, can mitigate delayed testicular development and impaired semen quality, but studies have shown that some patients still do not return to baseline semen quality after surgery (Arab et al., 2021; Shomarufov et al., 2022). There is an urgent need to find effective complementary alternative therapies. Since the etiology of VC-induced male infertility is complex and cannot be treated by single-target therapy, the multi-target treatment model of traditional Chinese medicine (TCM) is more suitable for the treatment of varicocele-associated male infertility. In recent years, the use of TCM has achieved better efficacy in the treatment of varicocele-associated male infertility, and some botanical preparations are gradually being applied against varicocele-associated male infertility (Dun et al., 2015; Nimrouzi and Zarshenas, 2016; Lu et al., 2020).

Mailuoshutong pill (MLST), a traditional Chinese herbal formula, each Gram of MLST is made from *Astragalus mongholicus* Bunge (Fabaceae, 0.833 g), *Lonicera japonica* Thunb (Caprifoliaceae, 0.833 g), *Phellodendron amurense* Rupr (Rutaceae, 0.417 g), *Atractylodes lancea* DC (Asteraceae, 0.417 g), *Coix lacryma-jobi* L (Poaceae, 0.833 g), *Scrophularia ningpoensis* Hemsl (Scrophulariaceae, 0.833 g), *Angelica sinensis* Diels (Apiaceae, 0.417 g), *Paeonia lactiflora* Pall (Paeoniaceae, 0.417 g), *Glycyrrhiza glabra* L (Fabaceae, 0.138 g), *Hirudo* (Hirudinidae, 0.417 g), *Scolopendra* (Scolopendridae, 0.033 g), *Scorpio* (Buthidae, 0.138 g). Pharmacological studies of TCM have shown that the active components of these herbs have biological activities of antioxidant, anti-inflammatory, blood circulation promotion, hormone regulation, and immune enhancement (Chen et al., 2010; Chen et al., 2011; Zhang et al., 2011; Chen et al., 2021). Clinical study has shown that MLST is effective in improving VC local clinical symptoms and secondary spermatogenic dysfunction (Liu and Hou, 2020). The effect of MLST against varicocele-associated male infertility and its mechanism is worthy of being studied. However, the mechanism of MLST for treating varicocele-associated male infertility is unclear due to its complex components and therapeutic targets. Therefore, an effective method is needed to decipher the relationship between MLST and varicocele-associated male infertility.

**FIGURE 1:**
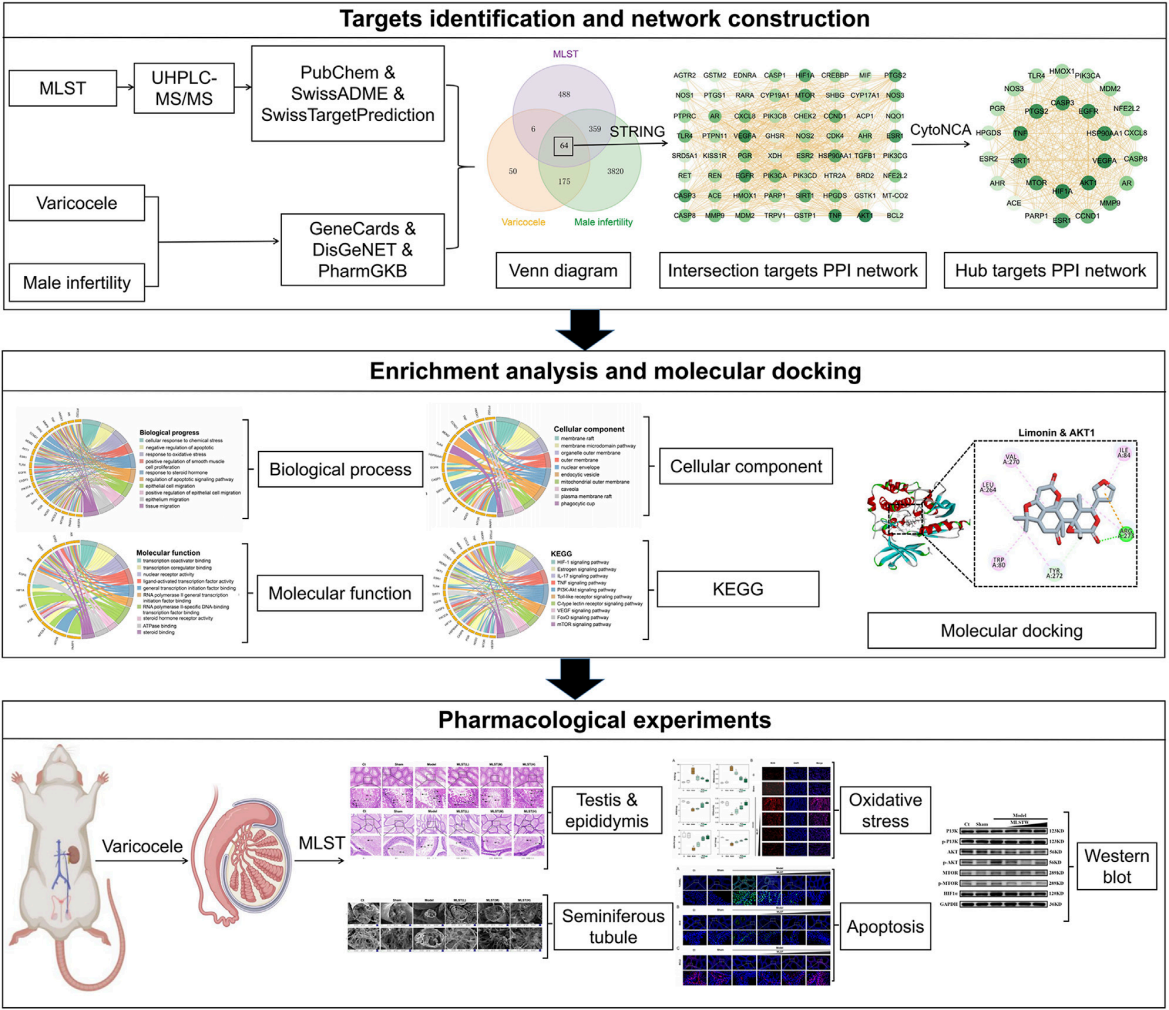
Flowchart for the network analysis and pharmacological experimental methods used in this study.

In this study, we used a combination of ultra-high-pressure liquid chromatography-mass spectrometry/mass spectrometry (UHPLC-MS/MS), network analysis, and pharmacological experiments to discover the phytochemical characterisation and multitarget mechanism of MLST against varicocele-associated male infertility. UHPLC-MS/MS was used to identify the main active components of MLST, and network analysis was used to construct the “Drug-Components-Targets-Disease” network to predict the potential drug components, drug targets, and drug mechanisms of MLST. Finally, after the prediction of network analysis, we conducted an experimental pharmacological evaluation based on the pathogenesis of varicocele-related male infertility to elucidate its therapeutic effects and possible mechanisms of action, which can help guide the further development and clinical application of MLST. The flowchart of this study is shown in Figure 1.

## Materials and methods

### Chemicals and instruments

MLST was provided by Shandong Lunan Pharmaceutical Group (Linyi, China). Methanol and acetonitrile were supplied by Merck KGaA at LC-MS purity, and formic acid was supplied by Sia Reagents at LC-MS purity. Malondialdehyde (MDA) colorimetric assay kit (eLabscience, E-BC-K025-M), superoxide dismutase (SOD) activity assay kit (eLabscience, E-BC-K022-M), catalase (CAT) activity assay kit (eLabscience, E-BC-K031-M), glutathione peroxidase activity assay kit (eLabscience, E-BC-K096-M), reduced glutathione colorimetric assay kit (eLabscience, E-BC-K030-M), reactive oxygen species (ROS) (Beijing Huaying Biotechnology Research Institute, HY-M0087), TUNEL test kit (Genepool, GPB 1829), BAX antibody (Bioss, bs-0127r), BCL2 antibody (Bioss, bs-33047 m), PI3K antibody (bioss, bs-10657r), p-PI3K antibody (bioss, bs-6417r), AKT antibody (bioss, bs-6951r), p-AKT antibody (bioss, bs-0876r), mTOR antibody (bioss, bs-1992r), p-mTOR antibody (bioss, bs-3495r), HIF1α antibody (bioss, bs-0737r), ECL kit (SUKER, ED0015-B). Ultra-performance liquid chromatography (Thermo Fisher Scientific, Q-Exactive HF), high-resolution mass spectrum (Thermo Fisher Scientific, Q-Exactive HF), column (Agilent technologies, Zorbax Eclipse C18), inverted fluorescence microscope (Japan’s Nikon, Nikon Eclipse Ti-SR), panoramic scanner (3D HISTECH, Pannoramic MIDI), scanning electron microscopy (SEM) (FEI Corporation of America, FEI Quanta250).

### MLST components identification

By using UHPLC-MS/MS to determine the components in MLST. Weigh 100 mg of MLST, add 1 ml of 70% methanol, shake and centrifuge, take the supernatant for dilution, 10 μl 100 μg/ml internal standard, pass through 0.22 μm PTFE filter tip on the machine for analysis, using ultra-performance liquid chromatography and high-resolution mass spectrum, the column for separation, separation conditions are column temperature of 30°C; flow rate of 0.3 ml/min; mobile phase composition A: water +0.1% formic acid, B: pure acetonitrile, the injection volume was 2 μl, and the autosampler temperature was 4°C.In this study, the identified components of MLST were further screened based on gastrointestinal absorption rate and drug similarity (ADME principles) using SwissADME online system (http://www.swissadme.ch/) (Daina et al., 2017). The components in MLST obtained from the screening were used for further studies.

### Network analysis, drug targets and disease targets screening

SwissTargetPrediction online platform (http://www.swisstargetprediction.ch/) (Daina et al., 2019) is a web server that accurately predicts chemical component targets based on known ligands combined with 2D and 3D similarity measures. SwissTargetPrediction was used to predict the targets of the study component, and the species was set to “Homo sapiens,” and the targets with “probability >0” were selected for the follow-up study. The predicted targets were imported into an Excel sheet to create a database of MLST targets.

GeneCards (https://www.genecards.org/) (Stelzer et al., 2016) is a comprehensive human genetic database that includes all known and predicted human genes in terms of genome, proteome, transcription, and genetics. DisGeNET (https://www.disgenet.org/) (Pinero et al., 2020) is a general-purpose platform for studying the molecular basis of human diseases and their complications, validating disease candidate genes, and evaluating the performance of text mining methods. Pharmacogenetics and Pharmacogenomics Knowledge Base (PharmGKB) (https://www.pharmgkb.org/) (Barbarino et al., 2018) is the most authoritative and complete pharmacogenomic database containing information on how human genetic variation affects drug response. We searched the GeneCards database, DisGeNET database, and PharmGKB database for the targets related to varicocele-associated male infertility by using “varicocele” and “male infertility” as keywords, and recorded them in an Excel sheet.

### Protein-protein interaction network and drug-components-targets-disease network construction

Duplicate data of drug targets and disease targets were removed separately and imported into origin software (version 2021) to plot Venn diagrams, and the intersection targets were considered to be effective targets for MLST treatment of varicocele-associated male infertility. STRING (https://cn.string-db.org/) (Szklarczyk et al., 2019) can be used to identify interactions between target proteins. The intersection targets were imported into the STRING database, the species was set to “Homo sapiens,” and the interaction between the target proteins was constructed. The results of the PPI analysis were imported into Cytoscape software (Version 3.7.2), and topological analysis of the PPI network was performed using the CytoNCA plug-in to obtain comprehensive data of each node (Tang et al., 2015). According to the network analysis method (Liu et al., 2022), the hub targets of MLST for the treatment of varicocele-related male infertility were screened by setting the degree centrality greater than 2 times the median and the betweenness centrality and closeness centrality both greater than the median. The components-targets mapping relationships were imported into Cytoscape software to construct a “Drug-Components-Targets-Disease” network.

### Enrichment analysis of hub targets

Gene ontology (GO) and Kyoto Encyclopedia of Genes and Genomes (KEGG) enrichment analyses of hub targets were performed to reveal the role of identifying hub targets in varicocele-associated male infertility progression and treatment. Enrichment analysis of hub targets was performed using the clusterProfiler package (Wu et al., 2021) in R software. GO enrichment analysis annotates the hub targets’ function in terms of cellular component, biological process, and molecular function, and KEGG enrichment analysis describes the pathways of action of the hub targets. When *p* < 0.05, these targets were found to be significantly enriched in GO terms or KEGG pathways. The GOplot package (Walter et al., 2015) of R software was used to visualize the results of the GO and KEGG analyses.

### In silico molecular docking

The structures of the ligands were obtained from the PubChem database (https://pubchem.ncbi.nlm.nih.gov/) (Kim et al., 2016), and the structures of the receptors were obtained from the RCSB Protein Data Bank (https://www.pdbus.org/) (Burley et al., 2022). The protein crystal structures were imported into Discovery Studio (Version 2019) and water was removed, hydrogen atoms were added and incomplete residues were supplemented. Active pockets were defined based on the original ligands in the complex and molecular docking was performed by the CDOCKER algorithm to calculate the root mean square deviation (RMSD) of the co-crystallized ligands, where RMSD values below 2Å were considered as good solutions reflecting the reliability of the docking model, and the scoring of the -CDOCKER interaction energy (-CIE) was used to evaluate the ligand and receptor binding ability, and the original ligand-CIE was used as a positive control (Ramirez and Caballero, 2018). Finally, the ligand-receptor affinity was assessed by docking the active ingredient with key potential targets and calculating the-CIE compared with the original ligand, using the positive control score as the threshold and a drug score value > threshold × 75% as the criteria for strong ligand-receptor affinity.

## *In Vivo* pharmacological experiments

### Preparation of animal models and interventions

36 SD rats, aged 8 weeks (body weight, 240–260 g), were purchased from Beijing Viton Lever Laboratory Animal Technology Co., (Beijing, China). The rats were placed in an animal room under 22 ± 1°C and 60% ± 2% interior design conditions with a light/dark cycle for 12 h. All experimental procedures involving animals were approved by Institutional Animal Care and Use Committee of Shandong University of Traditional Chinese Medicine and Animal Ethics Committee of Affiliated Hospital of Shandong University of Traditional Chinese Medicine, Jinan, China (Approval Number: 2021-40). The animal care and use system and guidelines of Shandong University of Traditional Chinese Medicine were followed. Rats needed at least 1 week to adapt to the environment before doing the experiments.

In this study, the classical way of establishing a VC rat model through a narrow left renal vein created by Turner (Turner, 2001) was used. The rats were anesthetized intraperitoneally with 3% chloral hydrate (10 ml/kg), and after successful anesthesia, the rats were fixed, the skin was taken for positioning, dissected layer by layer, and the left abdominal contents were gently pushed to the right upper abdomen to fully reveal the left renal vein, and a 0.85 mm diameter metal probe was placed under the left renal vein, and after the 4–0 silk ligated the renal vein, the probe was withdrawn, resulting in local stenosis of the left renal vein. The left kidney was observed for approximately 2 min and it was confirmed that the organ was not significantly ischemic. In the sham-operated group, only the left renal vein was isolated and not ligated. The experimental diagram of the rat spermatic varicocele model construction is shown in Figure 4A. The criteria for the success of the model: the left spermatic vein is significantly tortuous and dilated compared with the right, and the left kidney has no atrophy.

One week after modeling, 36 rats were randomly divided into the following six groups (6 rats in each group): a normal control group (Ct), a sham-operated group (Sham), a VC-induced model group (Model), a VC-induced group administrated with low-dose MLST, a VC-induced group administrated with medium-dose MLST, and a VC-induced group administrated with high-dose MLST. The dose of MLST intervention in rats was determined based on the human equivalent dose, which was calculated from the body surface area equivalent dose (Blanchard and Smoliga, 2015). The low-dose MLST group, medium-dose MLST group, and high-dose MLST group were gavaged with MLST 0.162 g/kg, 0.324 g/kg, and 0.648 g/kg respectively, and MLST was dissolved in 2 ml sodium carboxymethyl cellulose (CMC-Na) solution during gavage. The Ct group, Sham group, and model group were given 2 ml CMC-Na solution by gavage as control. After 30 days of intervention, the rats and left testes were weighed and the testis index was calculated, testis index (%) = (testis weight/body weight) ×1,000‰ (Wang et al., 2020). Take a piece of testicular tissue and epididymal tissue, and store them in formalin respectively for histological evaluation (HE) staining. Take a piece of testicular tissue and store it in glutaraldehyde at room temperature for SEM observation. Three pieces of testicular tissue were put into cryopreservation tubes and stored in liquid nitrogen for Western blot (WB) analysis, oxidative stress level detection, and apoptosis detection.

### Histological evaluation staining

HE staining was used to evaluate the degree of testicular and epididymal tissue damage and sperm quality in each group. The left testis and epididymis tissues of each group of rats were fixed with 4% paraformaldehyde, dehydrated in alcohol, transparently treated with xylene, embedded in paraffin, and serially sectioned on a microtome with a slice thickness of 4 μm. The 4 μm sections were dewaxed, dehydrated, stained with hematoxylin, and sealed with neutral gum. Images of testicular and epididymal tissue structures were observed and acquired under an inverted fluorescence microscope and panoramic scanner.

Observation of Spermatogenic Tubule by SEM

Testicular tissues were taken and gently rinsed with PBS, fixed at room temperature for 2 h with electron microscopy fixative, fixed at room temperature for 2 h with 0.1 M phosphate buffer PB (PH 7.4) prepared with 1% osmium acid, and rinsed 3 times with 0.1 M phosphate buffer PB. The tissues were sequentially dehydrated with alcohol and isoamyl acetate. The samples were dried and placed tightly on the double-sided adhesive of conductive carbon film on the sample stage of the ion jetting instrument for the 30 s. Finally, the structure of the spermatogenic tubule was observed by SEM.

### Measurement of oxidative stress biomarkers

The levels and activities of MDA, GSH, SOD, CAT, and GSH-Px were measured with kits according to the instructions. Determination of ROS content by DHE fluorescence staining. Fresh testicular tissue was rewarmed in frozen sections, and the tissue was incubated with DHE diluted in PBS for 30 min at 37°C under light-proof conditions, then the slides were washed in PBS (PH 7.4) on a decolorized shaking table, and the sections were shaken dry, and then the nuclei were stained with DAPI staining solution for 10 min at room temperature and light-proof. The slides were then placed in PBS and washed three times for 5 min each time on a decolorized shaking table with no light, and the sections were shaken dry and sealed with an anti-fluorescence quenching sealer. The slides were placed under an inverted fluorescence microscope and panoramic scanner, and images were collected to determine the amount and variation of ROS content in the cells according to the red fluorescence in the cells.

### TUNEL Staining

The testicular tissue was fixed with 4% paraformaldehyde, dehydrated, embedded, sectioned, dewaxed, added with fluorescence quenching agent for 5 min, washed with running water for 10 min, and the slides were treated according to the instructions of the TUNEL test kit (fluorescence method). After operating according to the instructions, wash with PBS (PH 7.4). After the slices are slightly dried, they are sealed with anti fluorescence quenching sealing agent (including DAPI). Slices were observed in the panoramic scanner and images were collected.

### Immunofluorescence

Testicular tissue was fixed in 4% paraformaldehyde, dehydrated, embedded, sliced, and washed with PBS (10X). The slices were placed in the repair box of EDTA (PH 8.0) antigen repair buffer, and the antigen was repaired in the steamer. The fluorescent quenching agent was added, washed with running water, and incubated with 5% BSA at room temperature. After discarding the serum, the primary antibody (1:100) was incubated overnight. After the slices were washed and dried with PBS, they were covered with a suitable kind of secondary antibody, dripped with DAPI dye, and sealed with anti fluorescence quenching sealant. Bax and BCL2 were selected for the primary antibody The staining of Bax and BCL2 in testicular tissue was observed by the panoramic scanner.

### Western blot analysis

Rat testicular tissue samples were ground into a powder with liquid nitrogen, lysed at 1 mg plus 10 μl of lysis solution for 30 min on ice, and the supernatant was collected by centrifugation at 4°C for 10 min after lysis. Protein (20 µg) was electrophoresed on 8%–12% SDS-PAGE gels, transferred to PVDF membranes, blocked with 5% BSA for 1 h at room temperature, and incubated with the following primary antibodies: PI3K, p-PI3K, AKT, p-AKT, mTOR, p-mTOR, HIF1α, diluted according to the antibody instructions. The primary antibody was incubated overnight at 4°C. The membrane was washed 5 times with TBST buffer for 5 min each time, and the goat anti-rabbit antibody was diluted with 5% BSA (1:5,000) according to the antibody instructions, mixed well, and the secondary antibody was incubated at room temperature for 1 h. The membrane was washed 5 times with 1 × TBST buffer for 5 min each time, and the ECL assay was performed using the ECL kit. Band intensity was analyzed by ImageJ software.

### Statistical analysis

All quantitative data were expressed as mean ± standard error of mean and statistically analyzed using GraphPad Prism 9 (GraphPad Software, San Diego, CA, United States ), and one-way analysis of variance (ANOVA) was used for comparison between groups. A difference of *p* < 0.05 was considered statistically significant.

## Results

### Potential components and targets of MLST on varicocele-associated male infertility

**FIGURE 2:**
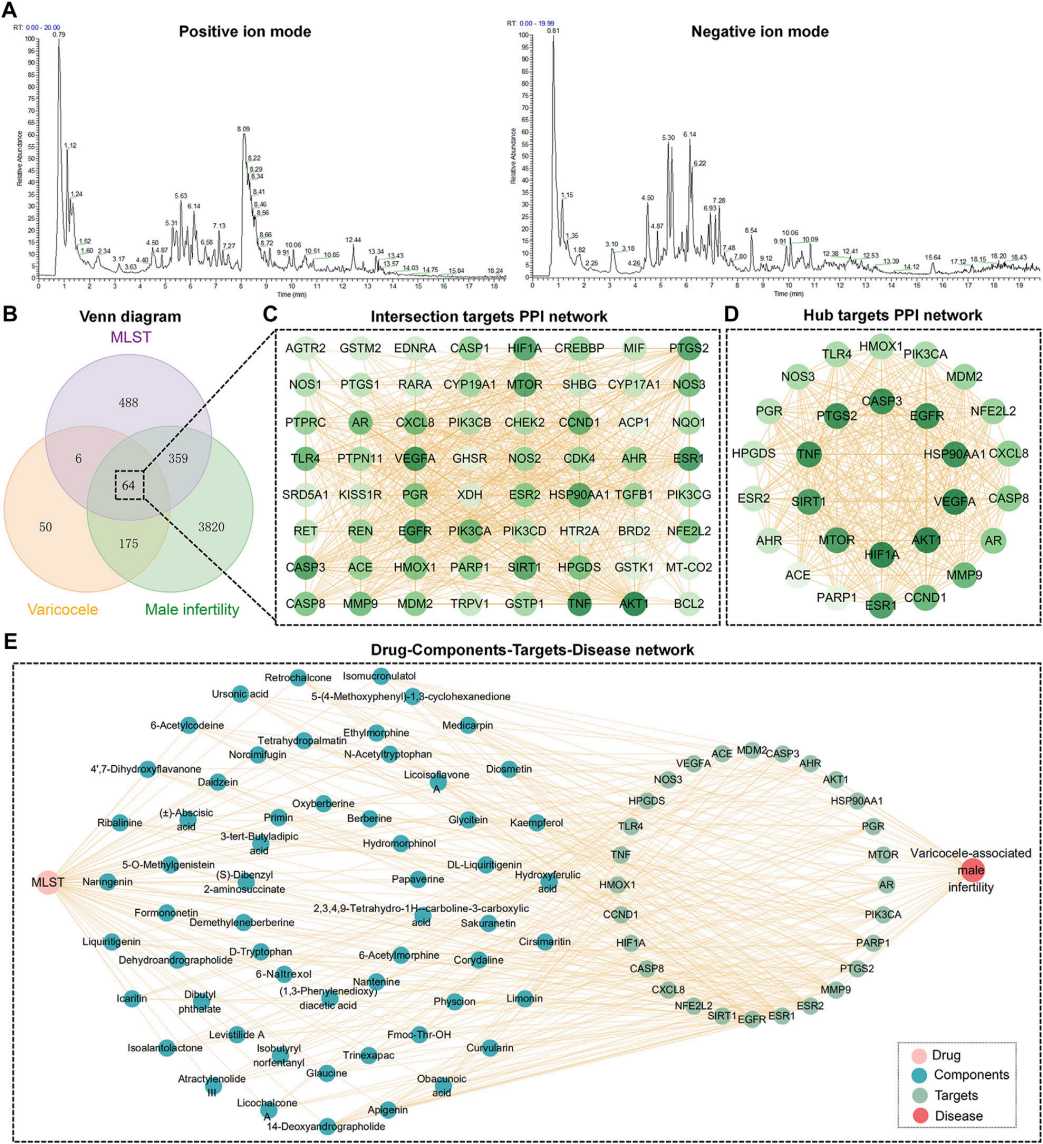
Network analysis of MLST against varicocele-associated male infertility. **(A)** Total ion flow diagram of components in MLST analyzed by UHPLC-MS/MS, including positive and negative ion modes. **(B)** Venn diagram of potential targets in MLST for varicocele-associated male infertility. **(C,D)** Intersection targets and hub targets PPI network, the darker the color of the node, the larger the degree value. **(E)** Drug-Components-Targets-Disease network.

Through UHPLC-MS determined, a total ion flow spectrum of the components in MLST was obtained (Figure 2A), and a total of 346 components were determined by comparison with the secondary mass spectrometry data. Through SwissADME online system, setting the gastrointestinal absorption rate as “high” and drug similarity as “yes,” 62 components were screened for future study (Supplementary Table S1).

Prediction of the targets of the included components by SwissTargetPrediction yielded a total of 62 components acting on 917 targets. Targets for varicocele and male infertility were searched in the GeneCards database, DisGeNET database, and PharmGKB database, and a total of 4,418 male infertility targets and 295 varicocele targets were obtained after removing duplicate entries. Finally, 64 intersecting targets were obtained by making a Venn diagram (Figure 2B). These 64 intersecting targets were imported into STRING for PPI analysis, and 665 pairs of interactions were obtained and visualized using Cytoscape software (Figure 2C). The analysis was performed by Cytoscape software, and 28 hub targets were obtained by filtering based on degree centrality, betweenness centrality, and closeness centrality. 28 hub targets were imported into STRING for PPI analysis and visualized using Cytoscape (Figure 2D).

The information of components and hub targets was imported into Cytoscape to construct the “Drug-Components-Targets-Disease” network, as shown in Figure 2E. The topological structure of the network was assessed with the network analyzer function of Cytoscape, and the node importance was expressed in terms of the degree. The top 10 components about degree were 14-Deoxyandrographolide, apigenin, cirsimaritin, kaempferol, obacunoic acid, curvularin, limonin, hydroxyferulic acid, medicarpin, and diosmetin.

### GO and kyoto encyclopedia of genes and genomes enrichment analysis of hub targets

**FIGURE 3:**
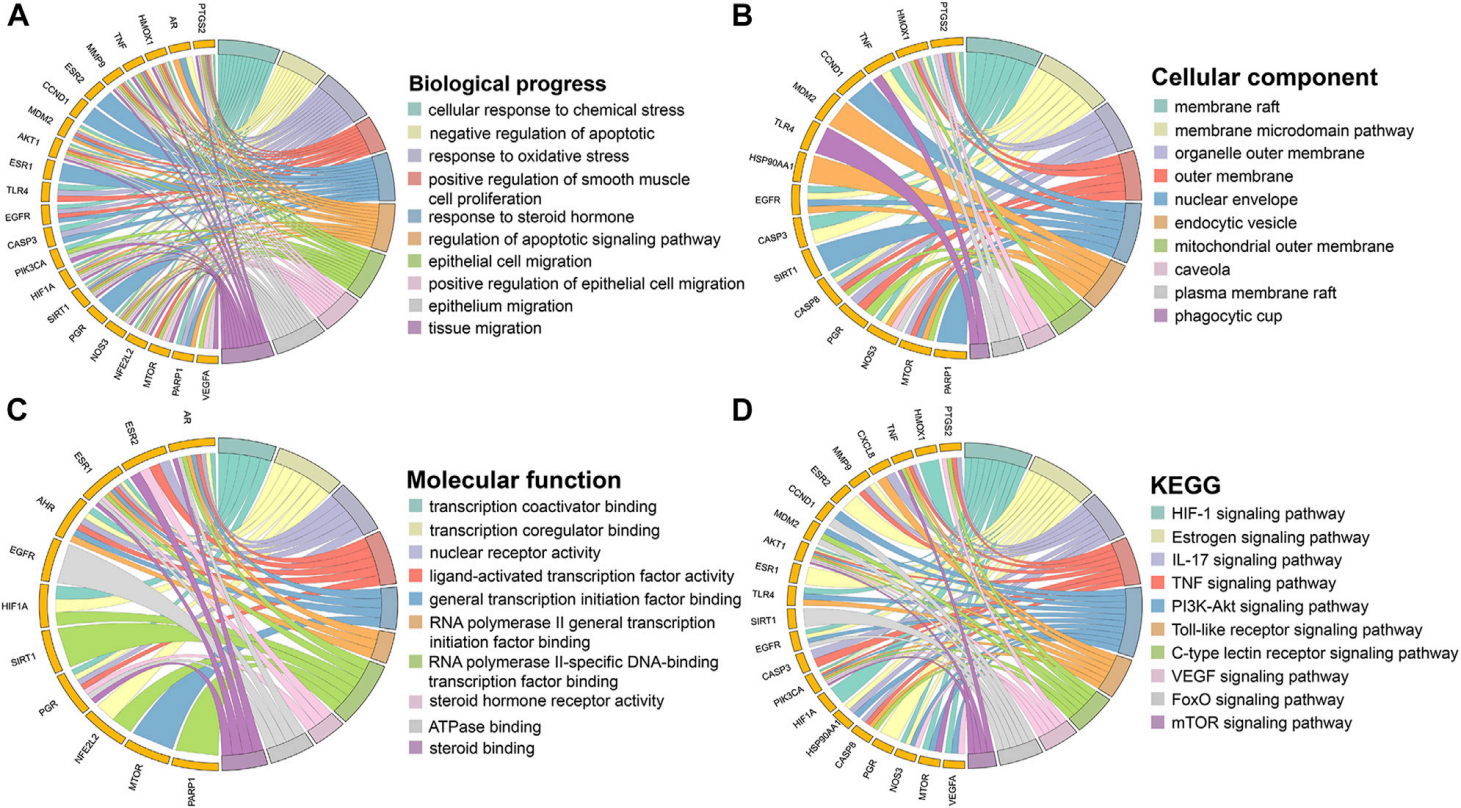
Enrichment analysis of hub targets. **(A)** Biological process. **(B)** Cellular component. **(C)** Molecular function. **(D)** KEGG.

GO enrichment analysis yielded 1,485 significant results, including 1,422 BPs, 17 CCs, and 46 MFs. In the BP category, the main biological processes in which the core targets are involved are cellular responses to chemical stress, responses to oxidative stress, and regulation of apoptotic signaling pathways. In the CC category, the core targets were mainly enriched in cellular components such as membrane rafts, membrane microdomains, and organelle outer membranes. In the MF category, the core targets mainly play molecular functions such as transcriptional cofactor binding, transcriptional coregulator binding, and nuclear receptor activity. A total of 120 pathways were obtained by KEGG enrichment analysis. Signaling pathways for cancer and other diseases were excluded, and the top 10 pathways of high significance were selected based on their *p*-values. These signaling pathways included HIF-1 signaling pathways, IL-17 signaling pathways, TNF signaling pathways, PI3K/Akt signaling pathways, and mTOR signaling pathways. The top 10 entries in each category of biological process, cellular component, molecular function, and KEGG signaling pathways were screened based on *p*-value and plotted in a circle diagram in Figure 3.

### MLST relieves testicular and epididymal tissue damage

**FIGURE 4:**
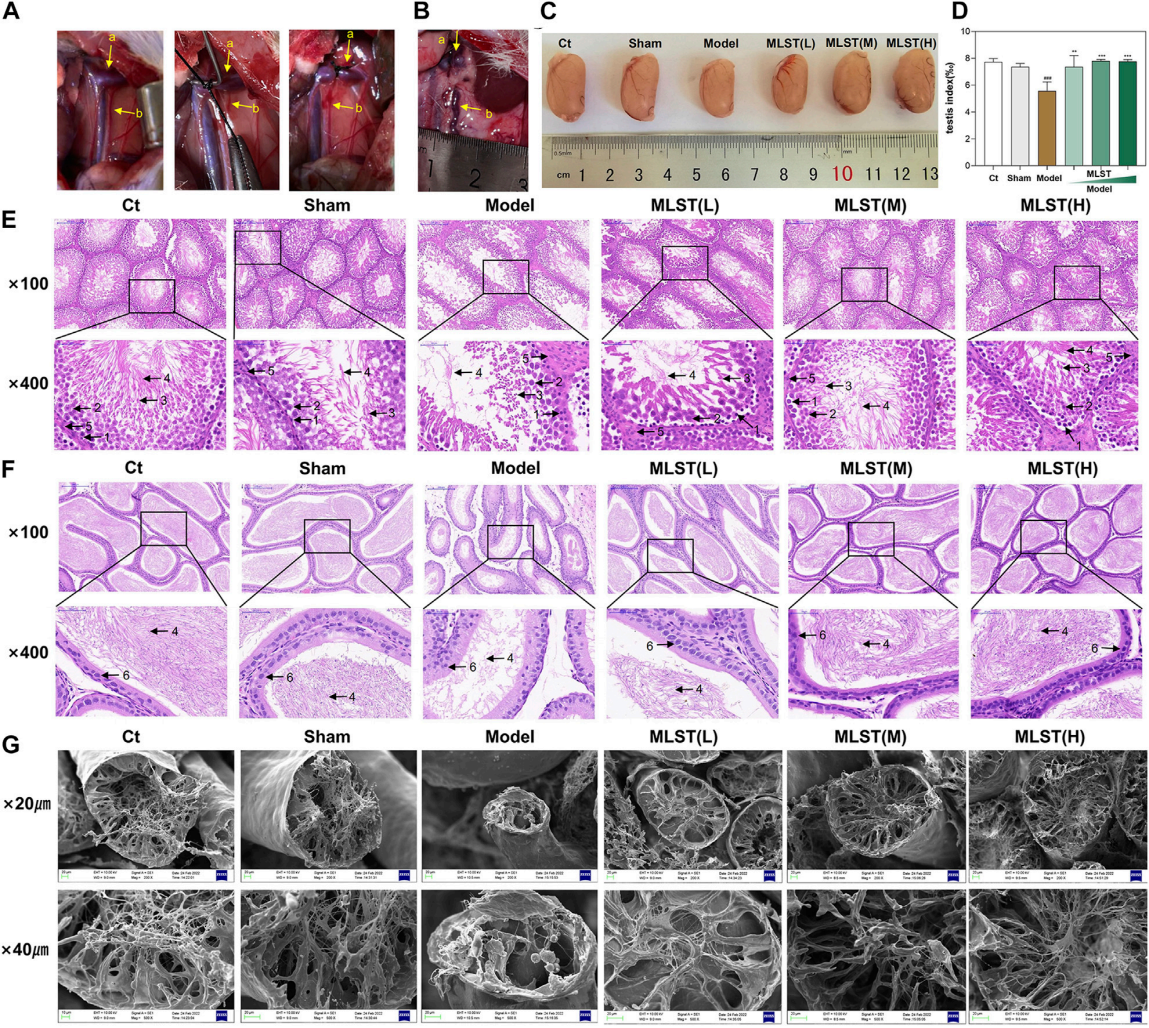
Preparation of VC rat model and histopathological changes of testicular tissue and epididymis. **(A)** The model of varicocele was made by narrowing the left renal vein and increasing the reflux pressure of the spermatic vein; a, left renal vein; b, spermatic vein. **(B)** The rat model of VC was successfully prepared by Turner’s method, and obvious varicocele was seen. **(C)** Testis of different groups. **(D)** Changes in testicular mass in different groups; N = 6; ^#^ *p* < 0.05, ^##^ *p* < 0.01, ^###^ *p* < 0.001 compared to Ct; *p < 0.05, **p < 0.01, ***p < 0.001 compared to Model. **(E)** HE staining of testicular tissue; 1, spermatogonia; 2, spermatocyte; 3, spermatid; 4, sperm; 5, leydig cell; (original magnification × 100, × 400). **(F)** HE staining of epididymal tissue; 4, sperm; 6, epithelial cell; (original magnification × 100, × 400). **(G)** Structural changes in the seminiferous tubules (scale bar × 20 μm, × 40 µm).

The VC rat model was successfully induced by narrowing the left renal vein, as shown in Figure 4B. The testis index of the rats in the model group decreased compared with the control group and the sham-operated group (*p* < 0.05), while the testis index of the rats with MLST intervention was better than that in the model group (*p* < 0.05) (Figures 4C,D).

Histopathological changes in testes and epididymis were observed by HE staining. As shown in Figure 4E, the testicular tissues of the control group were relatively intact, the germinal ducts were structurally sound and consisted of composite epithelium, and the spermatogonia, spermatocyte, and sperm, and, and leydig cells were neatly arranged, and there were a large number of sperm. Compared with the control group, the testicular tissue structure and morphology of the mice in the model group were changed, the spermatogonia, spermatocyte, and spermatid in the germinal ducts were disorganized and reduced in number, and the number of sperm was significantly reduced. Compared with the model group, the morphological structure of the testicular germinal ducts of rats in the low-dose MLST group was significantly improved, and the number of spermatogonia, spermatocyte, and spermatid was increased and arranged in a relatively orderly manner, and the number of sperm in seminiferous tubule was increased. The testicular tissue morphology of the medium-dose MLST group and high-dose MLST was similar to that of the control group. As shown in Figure 4F, the epithelial cells of the epididymal duct lumen in the control and sham-operated groups were neatly arranged, which contained a large number of normal sperm. In the model group, the epididymal duct lumen was atrophied, the arrangement of the epithelial cells was disordered, and the density of sperm in the lumen of the epididymal duct was reduced. Compared with the model group, the morphological structure of the epididymal ducts in the low-dose MLST group was significantly improved, with epithelial cell arrangement and increased sperm density. The morphology, structure, and sperm density of the epididymal ducts in the medium-dose MLST group and high-dose MLST group were similar to those in the control group.

Furthermore, the damage of VC on the seminiferous tubule was further observed by scanning electron microscopy, and the protective effect of MLST on the seminiferous tubule was observed. As shown in Figure 4G, the structure of the seminiferous tubule in the control and sham-operated groups was normal. In the model group, the seminiferous tubule of rats was narrowed and concentrated, the number of spermatozoa tails was significantly reduced, supporting irregular arrangement, only a small number of spermatozoa were visible, and the lumen of the tubules was reduced. In the low-dose MLST group, the structure of the seminiferous tubules was improved, and an increase in the number of tubule lumens and normal spermatozoa was visible. The morphological structure of the seminiferous tubule in the medium-dose MLST group and high-dose MLST group was similar to that of the normal group. These results suggest that MLST ameliorates VC-induced impairment of spermatogenic function.

### MLST relieves varicocele-induced oxidative stress

**FIGURE 5:**
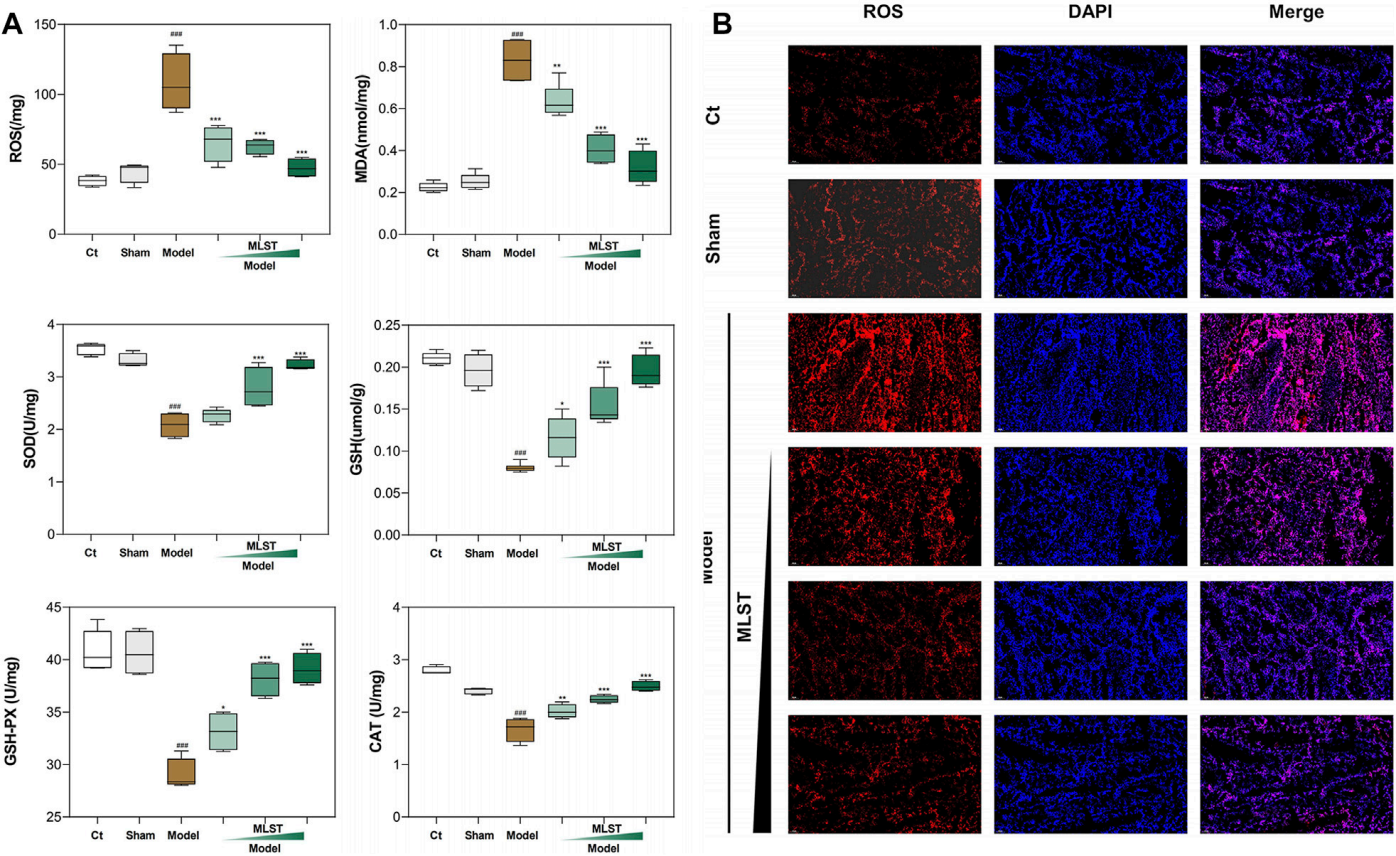
Effect of MLST treatment on VC-induced biomarkers of oxidative stress in rat testis. **(A)** Oxidative stress biomarkers, including ROS, MDA, SOD, GSH, GSH-Px, CAT; N = 6; ^#^ *p* < 0.05, ^##^ *p* < 0.01, ^###^ *p* < 0.001 compared to Ct; *p < 0.05, **p < 0.01, ***p < 0.001 compared to Model. **(B)** ROS fluorescence analysis; Red fluorescence represents ROS; Blue fluorescence represents cell nucleus.

The results of ROS, MDA, and antioxidant enzymes (SOD, GSH, GSH-Px, and CAT) in testicular tissues are shown in Figure 5A. The levels of MDA and ROS in the model group were significantly higher than those in the control and sham-operated groups (*p* < 0.05). MLST reduced the levels of ROS and MDA in testicular tissues of rats with VC, and the higher the dose, the more significant the effect. The levels of testicular antioxidant enzymes (SOD, GSH, GSH-Px, and CAT) were significantly decreased in the model rats compared with the control and sham-operated rats (*p* < 0.05). The levels of these enzymes were significantly increased in the rats with VC treated with MLST (*p* < 0.05), and the effect was more pronounced with higher doses. In terms of SOD parameters, the low-dose group showed increased values compared with the model group, but the results were not significantly different (*p* > 0.05).

In addition, ROS fluorescence analysis showed that the percentage of ROS expression in testis tissue was significantly higher in the model group than in the control and sham-operated groups, and MLST significantly reduced the ROS content in the testes of VC rats (Figure 5B). These results suggest that MLST can inhibit the VC-induced oxidative stress response.

### MLST relieves varicocele-induced apoptosis

**FIGURE 6:**
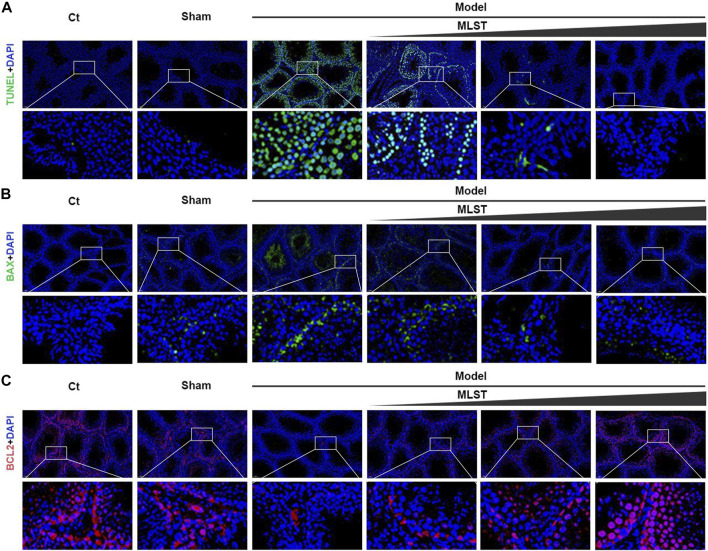
Effect of MLST on VC-induced apoptosis of rat testicular cells. **(A)** Green indicates TUNEL positive; Blue indicates cell nucleus stained by DAPI. **(B)** Green indicates DAPI staining of BAX; Blue indicates cell nucleus stained by DAPI. **(C)** Red indicates DAPI staining of BCL2; Blue indicates cell nucleus stained by DAPI.

TUNEL staining was used to detect apoptosis in testicular tissue. The number of TUNEL positive cells (green fluorescence) in the model group was significantly higher than that in the control group, while the number of TUNEL positive cells was significantly reduced by MLST intervention (Figure 6A). The expression of BAX and BCL2 in the testis was detected by immunofluorescence. For BAX (Figure 6B), the fluorescence intensity (green fluorescence) of the model group was significantly stronger than that of the control group, while the fluorescence intensity after MLST intervention was significantly lower than that of the control group. For BCL2 (Figure 6C), the fluorescence intensity (red fluorescence) of the model group was significantly lower than that of the control group, while the fluorescence intensity after MLST intervention was significantly higher than that of the control group. These findings suggest that MLST treatment can prevent VC-induced apoptosis in testicular tissue.

### Affinity of MLST core components to potential targets

**FIGURE 7:**
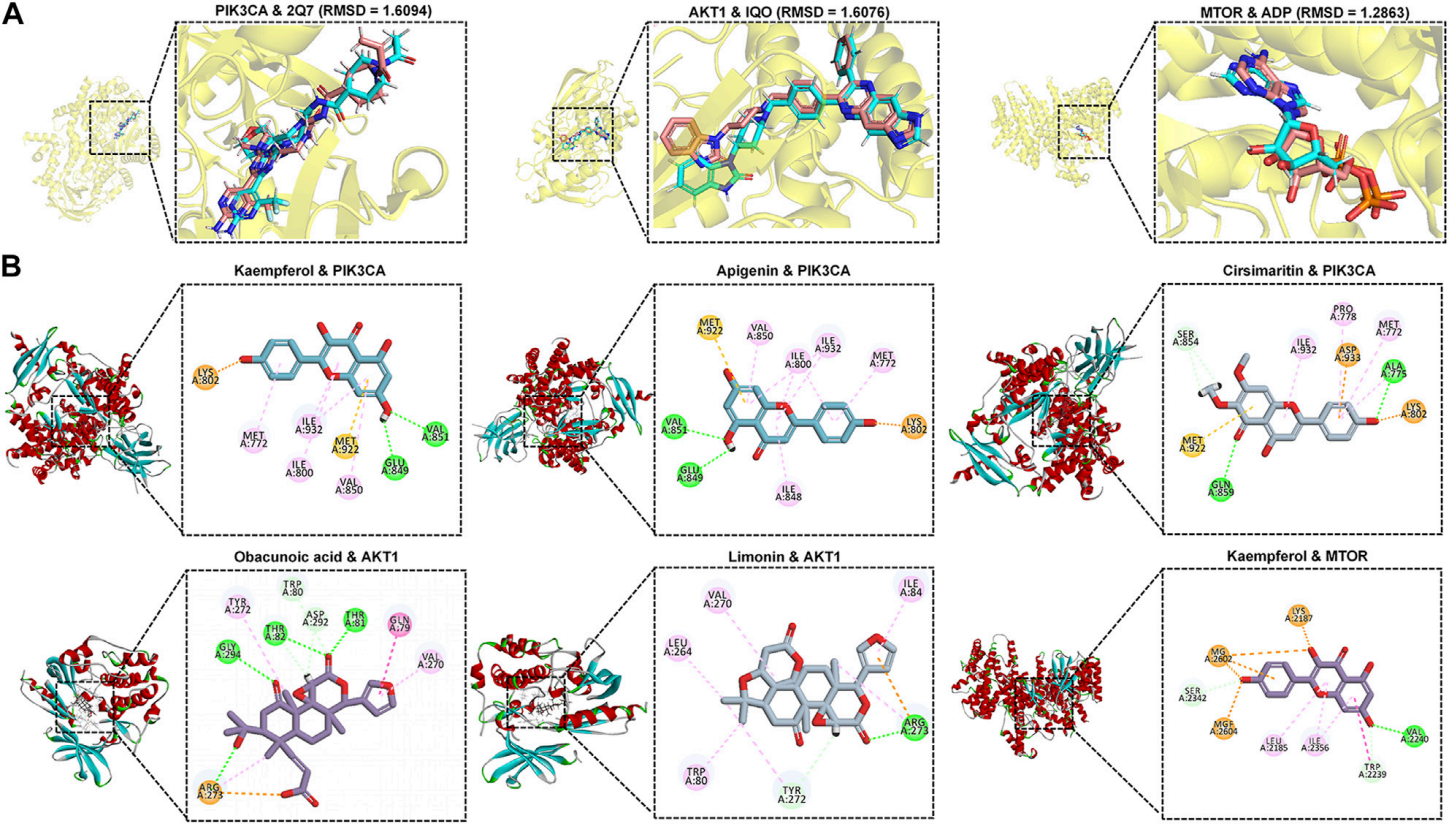
Molecular docking results of core components and potential targets. **(A)** RSMD of heavy ligands (blue) and co-crystalline ligands (pink) in selected target proteins (yellow). **(B)** Schematic diagram of the docking model of ligand-receptor molecules with strong affinity.

Hypoxia-inducible factor-1alpha (HIF1α) is crucial in varicocele-induced oxidative stress and apoptosis, and we found that PI3K/Akt, mTOR, and HIF-1 signaling pathways play an important role in HIF1α expression by enrichment analysis and review of related literature (Babaei et al., 2021), and by constructing the hub targets PPI network, it was found that HIF1α upstream related targets PIK3CA, AKT1, MTOR may be potential core targets for MLST in treating varicocele-associated male infertility. The protein crystal complexes (7R9V, 3O96, 3JBZ) were selected as docking targets for PIK3CA, AKT1, and MTOR, respectively, and the original ligands (2Q7, IQO, ADP) were used as positive controls, respectively (Wu et al., 2010; Lau et al., 2016; Borsari et al., 2022). The initial conformation and re-docking results of the original ligands were shown in Supplementary Table S2. The initial conformation and redocked conformation of the original ligands are shown in Figure 7A, and their RMSD values were less than 2Å, which responded to the reliability of the docking model. The -CDOCKER interaction energy value of PIK3CA with 2Q7 was 67.076, AKT1 with IQO was 69.216, and MTOR with ADP was 94.204, as a positive control drug score. The positive control drug score was used as the threshold value, and the drug score value > threshold value × 75% was used as the criterion that the drug had a strong affinity with the ligand. The results of the drug score value for the molecular docking of the top 10 components mentioned above with PIK3CA, AKT1, and MTOR were shown in Supplementary Table 3. Finally, kaempferol (56.668), apigenin (54.973), and cirsimaritin (51.116) had a strong affinity with PIK3CA, obacunoic acid (54.880), limonin (55.787) had a strong affinity with AKT1 and kaempferol (73.040) had a strong affinity with MTOR, and the interaction diagrams of the above drug components with the target proteins are shown in Figure 7B. In summary, kaempferol, apigenin, cirsimaritin, obacunoic acid, and limonin in MLST have a strong affinity for the upstream target proteins of HIF1α (PIK3CA, AKT1, MTOR).

### MLST relieves testicular damage by regulating the PI3K/Akt/mTOR signaling pathways

**FIGURE 8:**
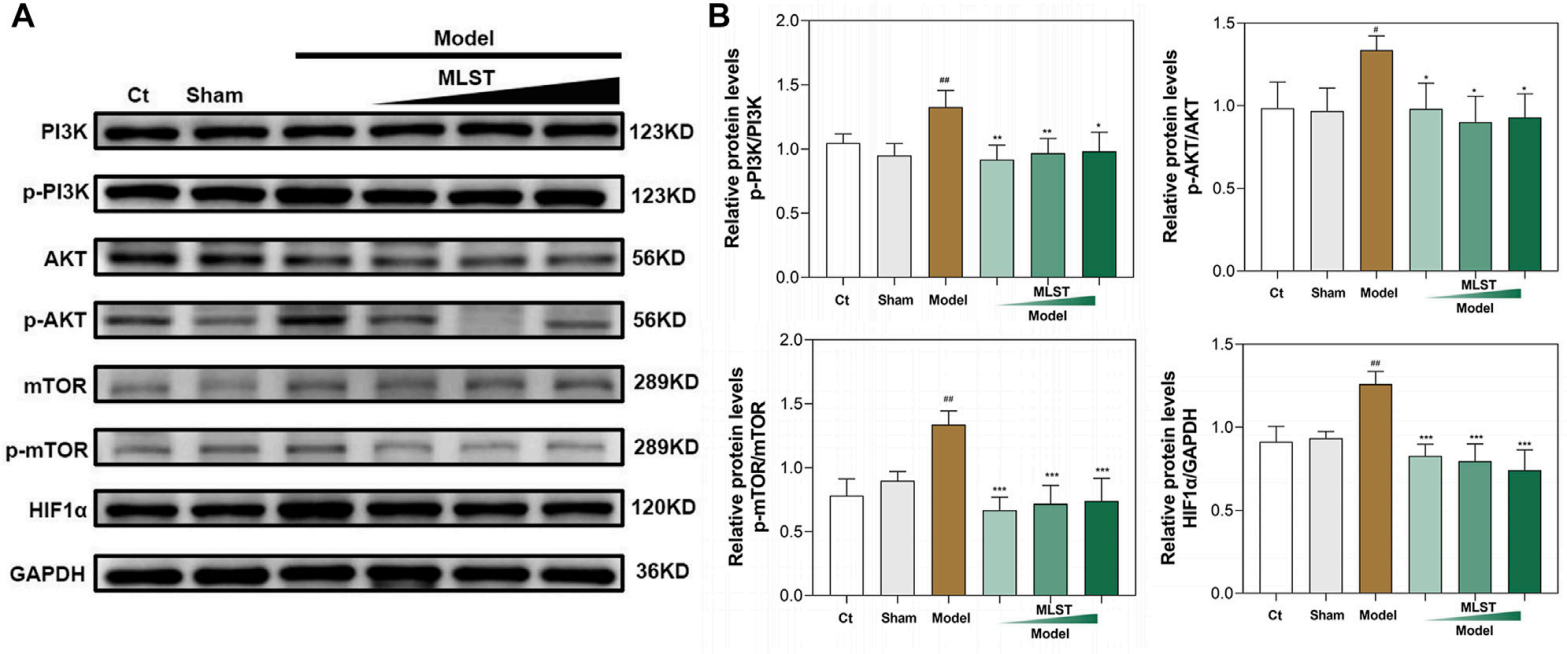
Results of western blots analysis of protein expression of PI3K/AKT/mTOR pathways. **(A)** Representative blot results of PI3K, p-PI3K, AKT, p-AKT, mTOR, p-mTOR, and HIF-1α. **(B)** Quantitative analysis of western blots for p-PI3K/PI3K, p-AKT/AKT, p-mTOR/mTOR, and HIF1α/GAPDH; N = 6; ^#^ *p* < 0.05, ^##^ *p* < 0.01, ^###^ *p* < 0.001 compared to Ct; *p < 0.05, **p < 0.01, ***p < 0.001 compared to Model.

As shown in Figure 8, by western blots analysis, HIF1α was significantly expressed in VC-induced rat testis compared to control (*p* < 0.05), and its upstream PI3K/Akt/mTOR signaling pathways-related protein (p-PI3K, p-AKT, p-mTOR) expression was increased (*p* < 0.05), indicating that VC activates the PI3K/Akt/mTOR signaling pathways and induces the expression of HIF1α. In contrast, HIF1α protein expression was reduced in MLST-treated rats compared with the model group (*p* < 0.05), and the expression of upstream PI3K/Akt/mTOR signaling pathways-related proteins (p-PI3K, p-AKT, p-mTOR) was reduced (*p* < 0.05), indicating that MLST inhibited the activation of PI3K/Akt/mTOR signaling pathways and reduced the expression of HIF1α.

## Discussion

VC is the main known cause of male infertility, and among male infertility patients with VC, there are varying degrees of abnormalities in total sperm count, sperm motility, and sperm morphology (Ni et al., 2016). Currently, semen quality can be improved and fertility improved in some patients through varicocelectomy (Lira Neto et al., 2021). However, some patients whose semen quality fails to improve after surgery, and the treatment effect is not satisfactory with the use of a single drug (Garg and Kumar, 2016; Yaris and Kilinc, 2022). The main reason for this is that the mechanism of male infertility caused by VC is complex and the use of a single drug, covering too few therapeutic targets, cannot play a better role. The multi-targeted therapeutic characteristics of TCM may be more applicable to the treatment of varicocele-associated male infertility (Qi et al., 2001). MLST is a Chinese patent medicine made by combining multiple Chinese medicines, and its “multicomponent, multitarget, multi-pathway” therapeutic characteristics have unique advantages over the use of other single drugs.

Firstly, we identified 62 components in MLST by UHPLC-MS/MS and ADME screening principles to provide component-based information on MLST. By constructing a “Components-Targets-Pathways” network and molecular docking, we screened the components with the strong binding ability to the pathway targets related to varicocele-associated male infertility: apigenin, kaempferol, limonin. Apigenin, as a biologically active flavonoid, has various biological properties (e.g., anti-inflammatory and antioxidant effects) and can reduce oxidative stress in the testis (Dang et al., 2017). Kaempferol is a natural flavonoid with a wide range of therapeutic properties such as antioxidant, anticancer and anti-inflammatory, and has been shown to promote spermatogenesis in VC-induced SD rats by modulating abnormal sex hormones, reducing oxidative stress, endoplasmic reticulum stress and apoptosis (Imran et al., 2019; Karna et al., 2019). Limonin is a natural tetracyclic triterpene compound with anti-inflammatory, analgesic, and antioxidant, and can reduce apoptosis by regulating the PI3K/Akt signaling pathway (Fan et al., 2019; Qiu et al., 2022). Although some of the components have been reported for the treatment of varicocele and male infertility, there are still some ingredients that have not been tested pharmacologically and need further validation.

Furthermore, the PPI network analysis was used to screen 28 hub targets of MLST against varicocele-associated male infertility. Among them, HIF1α, a highly specific nuclear transcription factor, is usually overexpressed in hypoxic environments and is an important hub for regulating oxygen homeostasis, and overexpression of HIF1α can induce testicular tissue damage (Kilinc et al., 2004). Vascular endothelial growth factor A (VEGFA), the most important vascular endothelial growth factor, plays an important role in regulating angiogenesis and formation and is involved in VC-induced testicular development and spermatogenesis (Shiraishi and Naito, 2008; Wang et al., 2017). Mammalian rapamycin (mTOR) is an important regulator of cell growth and proliferation, and studies have shown a positive correlation between mTOR expression and sperm DFI (Mahran et al., 2019). The results illustrated that MLST might regulate the above targets against varicocele-associated male infertility.

Furthermore, enrichment analysis of the hub targets revealed that MLST may regulate regulates oxidative stress and apoptosis to treat varicocele-associated male infertility. Oxidative stress is one of the main causes of testicular tissue damage induced by VC (Lai et al., 2022). Oxidative stress produces excessive ROS, which can not only lead to male infertility through lipid peroxidation or DNA damage but also lead to male infertility by inactivating enzymes and proteins in spermatogenesis (Wang et al., 2022). VC causes excessive ROS in testicular tissue, and can also cause testicular germ cell apoptosis, resulting in decreased spermatogenesis (Dolatkhah et al., 2020; Chakraborty and Roychoudhury, 2022).

**FIGURE 9:**
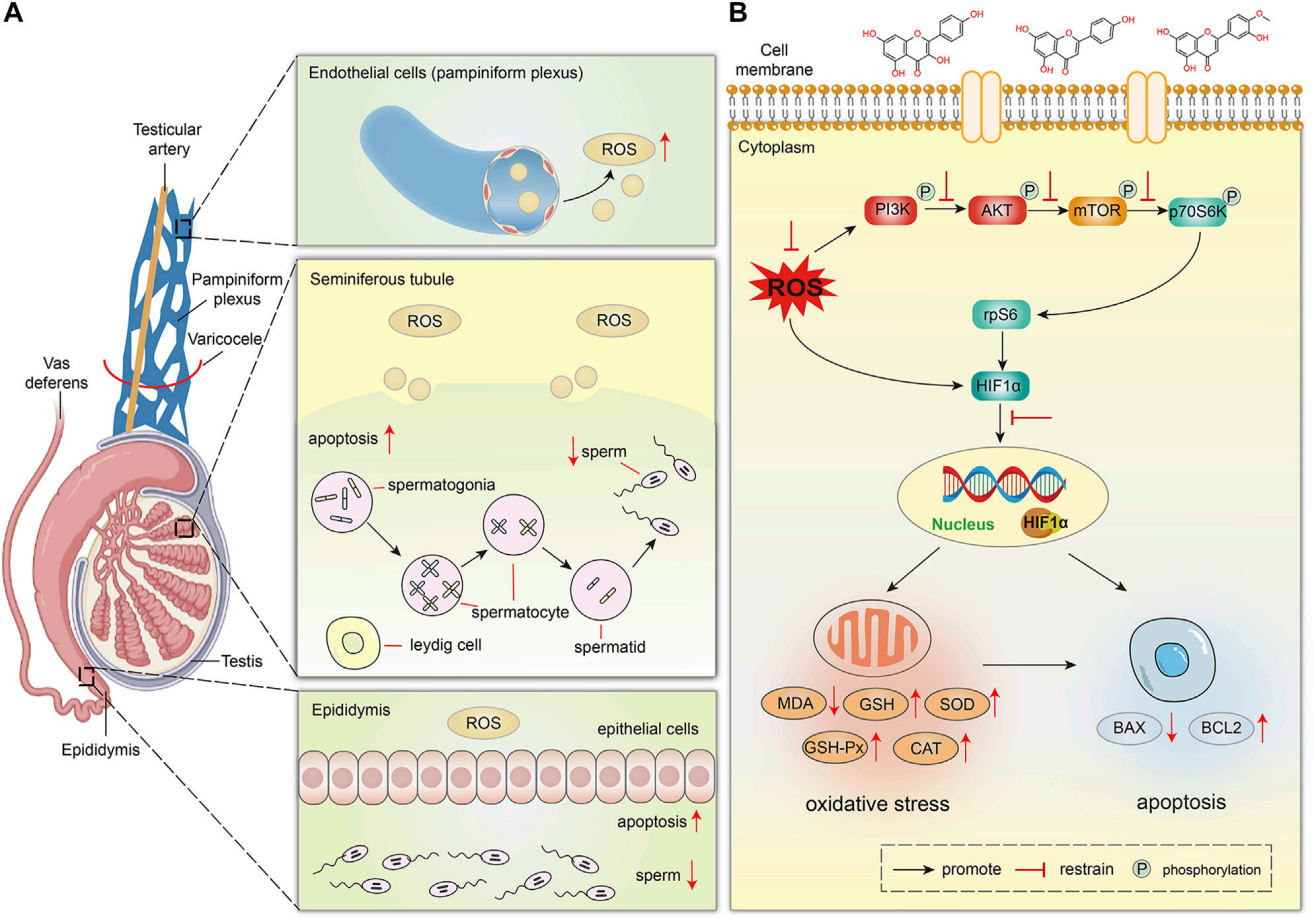
The proposed schematic diagram of MLST against varicocele-associated male infertility. **(A)** Effect of VC on testicular spermatogenesis in rats. **(B)** Mechanism of MLST against varicocele-associated male infertility.

Furthermore, we established a VC rat model to study the effect of VC on testicular spermatogenesis and the therapeutic effect of MLST on varicocele-associated male infertility. In this study, we found that VC can cause the decline of the testicular index, previous studies have also confirmed this phenomenon (Sakamoto et al., 2008), and MLST treatment can improve the testicular index of the VC rat model, indicating that MLST can improve VC-induced testicular dysplasia. In addition, VC-induced rats showed atrophy of seminiferous tubules and epididymal tubules, causing the loss and disorder of spermatogonia, spermatocyte, spermatid, leydig cell, and epithelial cell, resulting in impaired sperm, similar results have been reported in previous experimental studies of VC (Soni et al., 2018; Karna et al., 2019), these phenomena were significantly improved in rats treated with MLST, indicating that MLST can prevent VC-induced spermatogenic function damage. In this study, higher levels of MDA and ROS were detected in the testicular tissues of VC rats, while the activities of antioxidant enzymes, including SOD, GSH, GSH-Px, and CAT, were relatively low. The testicular tissue test results were similar to those of VC patients in the clinic (Dogan et al., 2014). The right amount of ROS is necessary for the maintenance of normal sperm function, and it can regulate sperm capacitation, acrosome reaction, and fusion with egg cells through intracellular signal transduction (Ford, 2004). Excessive ROS mediates the formation of MDA, which not only causes changes in cell membrane fluidity, and changes in the membrane composition of the sperm head and somatic cells, but also reacts with sperm DNA, causing DNA damage and changes (Serafini and O'Flaherty, 2021). TUNEL test showed that VC could cause apoptosis of testicular germ cells, and testicular germ cells affected sperm production (Kopalli et al., 2022). BAX and BCL2 fluorescence analysis also showed this result. In VC-induced rats, BAX expression increased and BCL2 expression decreased. After MLST treatment, testicular germ cell apoptosis decreased, BAX expression decreased, and BCL2 expression increased. It showed that MLST could treat varicocele-associated male infertility by inhibiting oxidative stress and apoptosis in testicular tissue. The effect of VC on testicular spermatogenesis is shown in Figure 9A.

Finally, HIF1α was overexpressed in VC-induced rat testes by western blot analysis, and excessive HIF1α expression could lead to impairment of testicular spermatogenesis, which was similar to previous reports (Lee et al., 2006), and MLST could reduce the impairment of testicular spermatogenesis by inhibiting HIF1α expression. Previous reports suggest that the expression of HIF1α is mainly affected by ROS and regulated by PI3K/Akt/mTOR signaling pathways (Babaei et al., 2021). On the one hand, excessive ROS can directly induce the expression of HIF1α, on the other hand, excessive ROS can activate the PI3K/Akt/mTOR signaling pathways, which can induce the expression of HIF1α. This was also confirmed in the present study that VC can lead to increased ROS expression in testicular tissue and activate the PI3K/Akt/mTOR signaling pathways. In contrast, in rats treated with MLST, the ROS content in testicular tissue was reduced and the PI3K/Akt/mTOR signaling pathways were also inhibited. The mechanism of MLST against varicocele-associated male infertility is shown in Figure 9B.

## Conclusion

The present study found that MLST was effective in alleviating VC-induced testicular tissue damage. The components in MLST were identified by UHPLC-MS/MS, which provided an important basis for the clarification of the material basis and subsequent network analysis. By predicting and elucidating the multi-component, multi-target, and multi-pathway therapeutic effects of MLST on varicocele-associated male infertility through network analysis, it was experimentally verified that MLST can inhibit the activation of the PI3K/Akt/mTOR signaling pathway, reduce the expression of HIF1α, and further attenuate VC-induced oxidative stress and apoptosis in the testis. These findings provide evidence for the therapeutic role of MLST in varicocele-associated male infertility, as well as an idea for the study of other herbal compounding.

## Data Availability

The original contributions presented in the study are included in the article/Supplementary Material, further inquiries can be directed to the corresponding author.
