# Supplementary material for: Mailuoshutong pill for varicocele-associated male infertility—Phytochemical characterisation and multitarget mechanism
Source: Front Pharmacol. 2022 Sep 9;13:961011. doi: 10.3389/fphar.2022.961011 (PMC9500298; doi:10.3389/fphar.2022.961011)
Supplement: Supplementary file 1 [file DataSheet1.docx]

Supplementary Material

Mailuoshutong pill for varicocele-associated male infertility – Phytochemical characterisation and multitarget mechanism

Dongfang Lv^1†^, Yun Ji^2†^, Qian Zhang^2^, Zhuozhuo Shi^2^, Tengfei Chen^2^, Chao Zhang^1^, Xiangyun Wang^1^, Taotao Ren^1^, Zhaowang Gao^2^, Chongfu Zhong^2*^

^1^College of First Clinical Medicine, Shandong University of Traditional Chinese Medicine, Jinan, China.

^2^Affiliated Hospital of Shandong University of Traditional Chinese Medicine, Jinan, China.

*** Correspondence:**Chongfu Zhong
zhongcf212@163.com

^†^These authors have contributed equally to this work and share first authorship.

Keywords: Mailuoshutong pill, varicocele-associated male infertility, phytochemical characterisation, multitarget mechanism, network analysis, experimental validation.

Table 1. MLST components identification by UHPLC-MC/MC and ADME principles.

| **NO.** | **Component name** | **Formula** | **Annotation MW** | **Calculated**  **MW** | **RT [min]** | **Structure** |
| --- | --- | --- | --- | --- | --- | --- |
| 1 | Boc-Asp-OH | C_9_H_15_NO_6_ | 233.08994 | 233.08989 | 0.814 |  |
| 2 | Isobutyryl norfentanyl | C_15_H_22_N_2_O | 246.17321 | 246.17323 | 2.487 |  |
| 3 | (1,3-Phenylenedioxy) diacetic acid | C_10_H_10_O_6_ | 226.04774 | 226.04737 | 3.896 |  |
| 4 | D-Tryptophan | C_11_H_12_N_2_O_2_ | 204.08988 | 204.08936 | 4.617 |  |
| 5 | Hydroxyferulic acid | C_10_H_10_O_5_ | 210.05282 | 210.0523 | 5.086 |  |
| 6 | (S)-Dibenzyl 2-aminosuccinate | C_18_H_19_NO_4_ | 313.13141 | 313.13116 | 5.346 |  |
| 7 | Ethylmorphine | C_19_H_23_NO_3_ | 313.16779 | 313.16756 | 5.404 |  |
| 8 | 2,3,4,9-Tetrahydro-1H-β-carboline-3-carboxylic acid | C_12_H_12_N_2_O_2_ | 216.08988 | 216.08988 | 5.498 |  |
| 9 | 6β-Naltrexol | C_20_H_25_NO_4_ | 343.17836 | 343.1782 | 5.814 |  |
| 10 | 6-Acetylcodeine | C_20_H_23_NO_4_ | 341.16271 | 341.16276 | 5.892 |  |
| 11 | 6-Acetylmorphine | C_19_H_21_NO_4_ | 327.14706 | 327.14695 | 6.029 |  |
| 12 | Nantenine | C_20_H_21_NO_4_ | 339.14706 | 339.14697 | 6.081 |  |
| 13 | Trinexapac | C_11_H_12_O_5_ | 224.06847 | 224.06836 | 6.128 |  |
| 14 | Sarracenin | C_11_H_14_O_5_ | 226.08412 | 226.084 | 6.382 |  |
| 15 | Papaverine | C_20_H_21_NO_4_ | 339.14706 | 339.14684 | 6.461 |  |
| 16 | Glaucine | C_21_H_25_NO_4_ | 355.17836 | 355.17832 | 6.562 |  |
| 17 | Kaempferol | C_15_H_10_O_6_ | 286.04774 | 286.04746 | 6.623 |  |
| 18 | Fmoc-Thr-OH | C_19_H_19_NO_5_ | 341.12632 | 341.12579 | 6.633 |  |
| 19 | 4',7-Dihydroxyflavanone | C_15_H_12_O_4_ | 256.07356 | 256.07335 | 6.715 |  |
| 20 | Hydromorphinol | C_17_H_21_NO_4_ | 303.14706 | 303.14679 | 6.779 |  |
| 21 | Demethyleneberberine | C_19_H_17_NO_4_ | 323.11576 | 323.11556 | 6.92 |  |
| 22 | N-Acetyltryptophan | C_13_H_14_N_2_O_3_ | 246.10044 | 246.10024 | 6.93 |  |
| 23 | Tetrahydropalmatin | C_21_H_25_NO_4_ | 355.17836 | 355.17837 | 6.964 |  |
| 24 | 5-(4-Methoxyphenyl)-1,3-cyclohexanedione | C_13_H_14_O_3_ | 218.09429 | 218.0939 | 7.171 |  |
| 25 | Diosmetin | C_16_H_12_O_6_ | 300.06339 | 300.06329 | 7.394 |  |
| 26 | Ribalinine | C_15_H_17_NO_3_ | 259.12084 | 259.12075 | 7.437 |  |
| 27 | Physcion | C_16_H_12_O_5_ | 284.06847 | 284.06825 | 7.442 |  |
| 28 | Corydaline | C_22_H_27_NO_4_ | 369.19401 | 369.1938 | 7.528 |  |
| 29 | Berberrubine | C_19_ H_15_NO_4_ | 321.10011 | 321.09988 | 7.806 |  |
| 30 | Norcimifugin | C_15_H_16_O_6_ | 292.09469 | 292.09442 | 7.975 |  |
| 31 | Daidzein | C_15_H_10_O_4_ | 254.05791 | 254.05782 | 8.043 |  |
| 32 | Berberine | C_20_H_17_NO_4_ | 335.11576 | 335.11521 | 8.211 |  |
| 33 | Sakuranetin | C_16_H_14_O_5_ | 286.08412 | 286.08383 | 8.375 |  |
| 34 | Oxyberberine | C_20_H_17_NO_5_ | 351.11067 | 351.11052 | 8.47 |  |
| 35 | (±)-Abscisic acid | C_15_H_20_O_4_ | 264.13616 | 264.1361 | 8.583 |  |
| 36 | 3-tert-Butyladipic acid | C_10_H_18_O_4_ | 202.12051 | 202.11994 | 8.677 |  |
| 37 | DL-Liquiritigenin | C_15_H_12_O_4_ | 256.07356 | 256.07353 | 8.751 |  |
| 38 | Glycitein | C_16_H_12_O_5_ | 284.06847 | 284.06847 | 8.924 |  |
| 39 | 5-O-Methylgenistein | C_16_H_12_O_5_ | 284.06847 | 284.06844 | 8.945 |  |
| 40 | Medicarpin | C_16_H_14_O_4_ | 270.08921 | 270.08923 | 9.248 |  |
| 41 | Retrochalcone | C_16_H_14_O_4_ | 270.08921 | 270.08931 | 9.665 |  |
| 42 | Naringenin | C_15_H_12_O_5_ | 272.06847 | 272.06855 | 9.724 |  |
| 43 | Apigenin | C_15_H_10_O_5_ | 270.05282 | 270.05252 | 9.757 |  |
| 44 | Liquiritigenin | C_15_H_12_O_4_ | 256.07356 | 256.07342 | 10.274 |  |
| 45 | Formononetin | C_16_H_12_O_4_ | 268.07356 | 268.07351 | 10.441 |  |
| 46 | Dehydroandrographolide | C_20_H_28_O_4_ | 332.19876 | 332.19866 | 10.584 |  |
| 47 | Obacunoic acid | C_26_H_32_O_8_ | 472.20972 | 472.20968 | 10.602 |  |
| 48 | Curvularin | C_16_H_20_O_5_ | 292.13107 | 292.13117 | 10.623 |  |
| 49 | Primin | C_12_H_16_O_3_ | 208.10994 | 208.10994 | 10.623 |  |
| 50 | Isomucronulatol | C_17_H_18_O_5_ | 302.11542 | 302.11517 | 10.732 |  |
| 51 | Limonin | C_26_H_30_O_8_ | 470.19407 | 470.19407 | 10.869 |  |
| 52 | Ursonic acid | C_30_H_46_O_3_ | 454.3447 | 454.34462 | 11.106 |  |
| 53 | Icaritin | C_21_H_20_O_6_ | 368.12599 | 368.12578 | 11.257 |  |
| 54 | Isoalantolactone | C_15_H_20_O_2_ | 232.14633 | 232.14629 | 11.472 |  |
| 55 | Cirsimaritin | C_17_H_14_O_6_ | 314.07904 | 314.07917 | 11.558 |  |
| 56 | Atractylenolide III | C_15_H_20_O_3_ | 248.14124 | 248.14114 | 11.649 |  |
| 57 | Licoisoflavone A | C_20_H_18_O_6_ | 354.11034 | 354.11012 | 11.731 |  |
| 58 | Licochalcone A | C_21_H_22_O_4_ | 338.15181 | 338.15148 | 11.746 |  |
| 59 | 14-Deoxyandrographolide | C_20_H_30_O_4_ | 334.21441 | 334.21431 | 11.881 |  |
| 60 | Dimefuron | C_15_H_19_ClN_4_O_3_ | 338.11457 | 338.1158 | 11.892 |  |
| 61 | Dibutyl phthalate | C_16_H_22_O_4_ | 278.15181 | 278.15167 | 14.007 |  |
| 62 | Levistilide A | C_24_H_28_O_4_ | 380.19876 | 380.1986 | 14.177 |  |

Table 2 The initial conformation and re-docking results of the original ligands.

| **Receptor** | **PDB: ID** | **Ligand** | **Pocket radius** | **Pocket site (x, y, z)** | **RSMD** | **-CDOKER interaction energy** |
| --- | --- | --- | --- | --- | --- | --- |
| PIK3CA | 7R9V | 2Q7 | 13.181 | -22.421, 10.476, 28.757 | 1.609 | 67.076 |
| AKT1 | 3O96 | IQO | 11.335 | 8.594, -7.042, 13.102 | 1.608 | 69.216 |
| MTOR | 3JBZ | ADP | 7.877 | -6.091, -38.095, -40.726 | 1.286 | 94.204 |

Table 3 The -CDOCKER interaction energy of the top 10 components mentioned above with potential core targets.

| **NO** | **Components** | **PIK3CA** | **AKT1** | **MTOR** |
| --- | --- | --- | --- | --- |
| 1 | 14-Deoxyandrographolide | 33.997 | 49.478 | 46.146 |
| 2 | Apigenin | 54.973 | 40.111 | 49.381 |
| 3 | Cirsimaritin | 51.116 | 50.806 | 62.238 |
| 4 | Curvularin | 31.006 | 39.994 | 45.725 |
| 5 | Diosmetin | 37.836 | 39.976 | 47.226 |
| 6 | Hydroxyferulic acid | 32.502 | 32.027 | 57.402 |
| 7 | Kaempferol | 56.668 | 51.991 | 73.040 |
| 8 | Limonin | 26.849 | 55.787 | 45.967 |
| 9 | Medicarpin | 29.180 | 36.770 | 33.760 |
| 10 | Obacunoic acid | 36.085 | 54.880 | 68.760 |

Table 4. Western blot images.

| P13K | GAPDH |
| --- | --- |
| 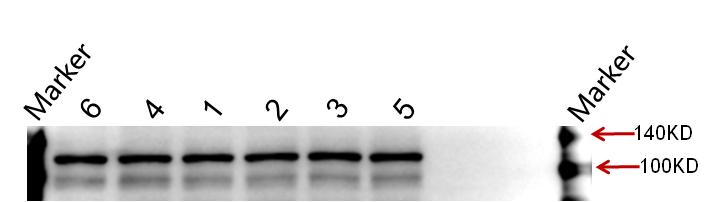 | 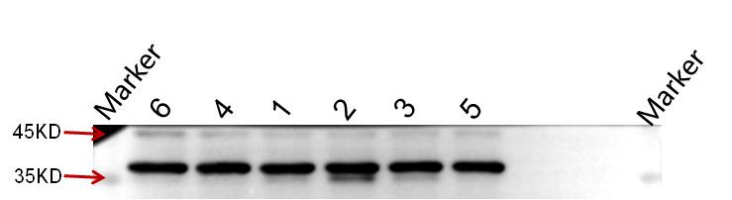 |
| p-PI3K | GAPDH |
| 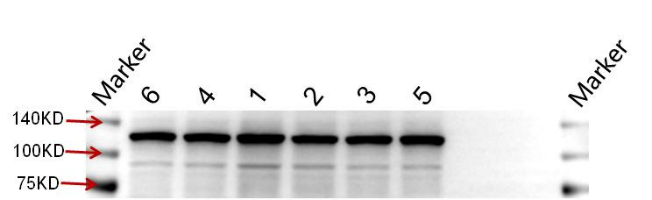 | 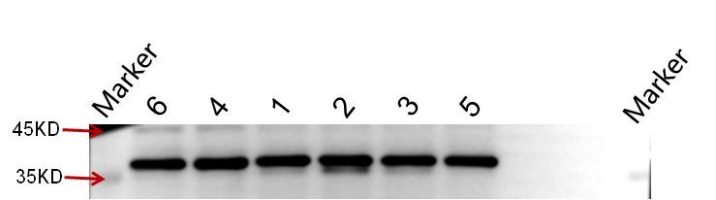 |
| 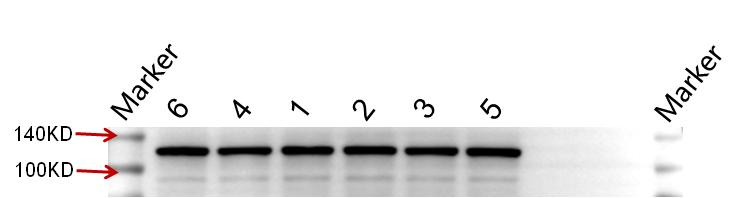 |  |
| 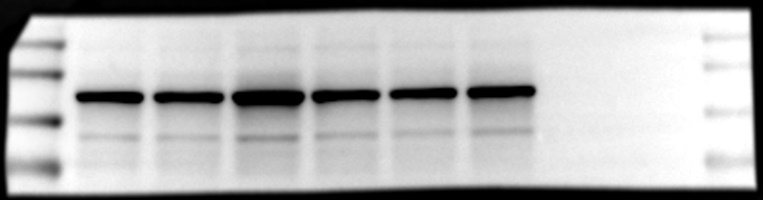 |  |
| AKT | GAPDH |
| 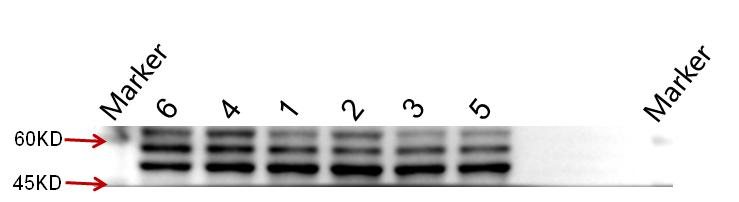 | 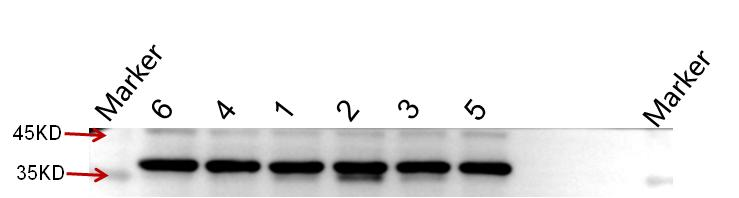 |
| P-AKT | GAPDH |
| 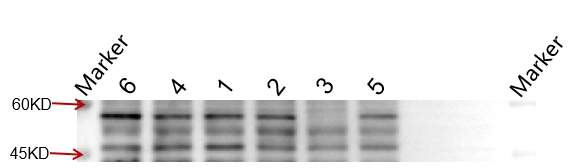 | 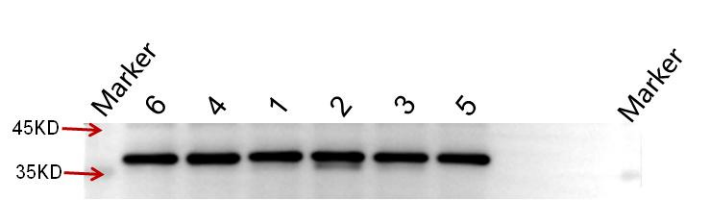 |
| 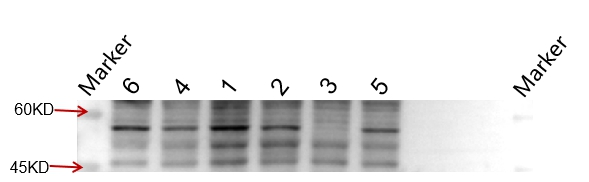 |  |
| 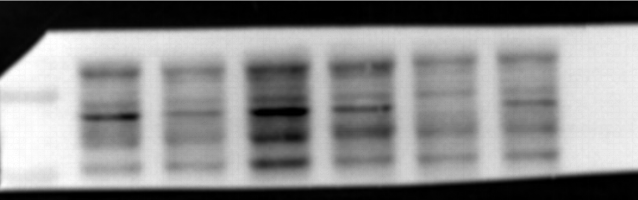 |  |
| MTOR | GAPDH |
| 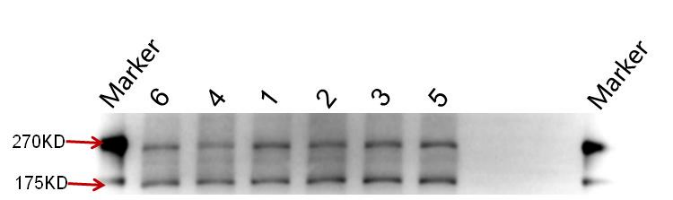 | 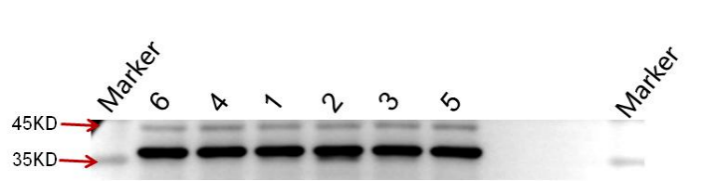 |
| p-MTOR | GAPDH |
| 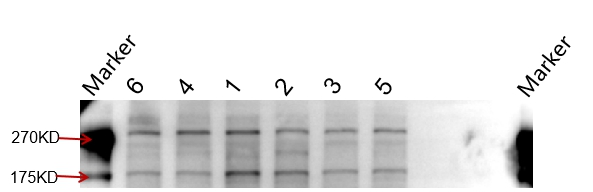 | 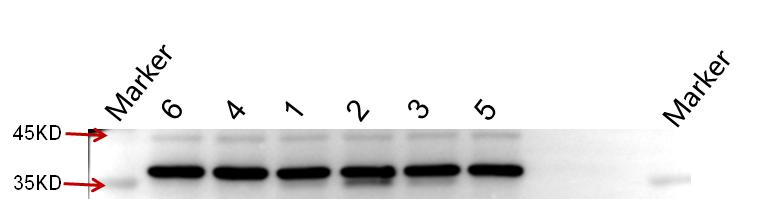 |
| 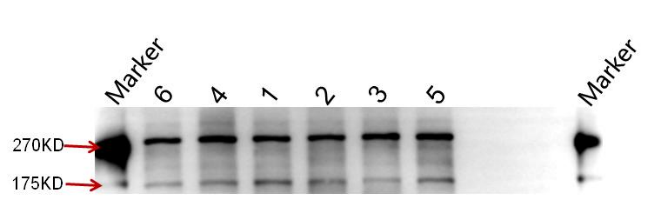 |  |
| 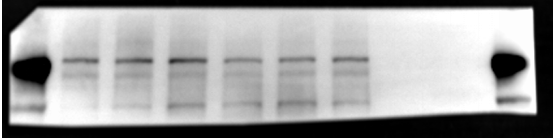 |  |
| HIF1α | GAPDH |
| 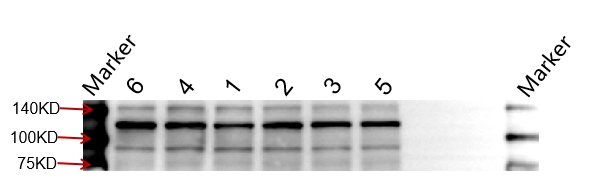 | 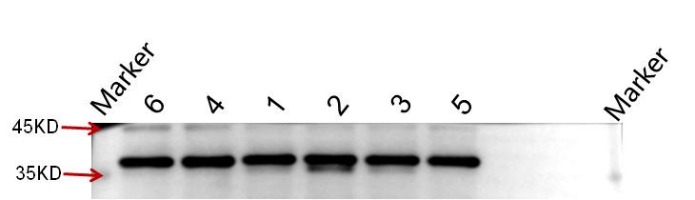 |
| 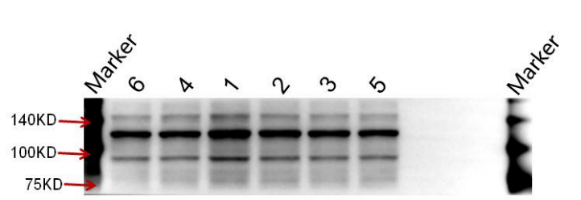 |  |
| 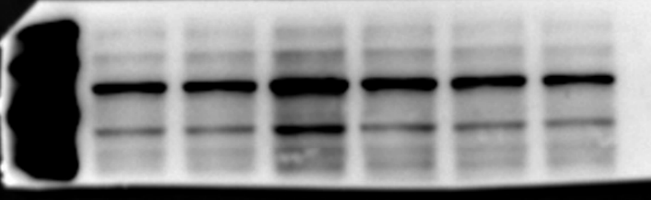 |  |
